# Weak transhumanism: moderate enhancement as a non-radical path to radical enhancement

**DOI:** 10.1007/s11017-023-09606-6

**Published:** 2023-02-13

**Authors:** Cian Brennan

**Affiliations:** University of Glasgow, Philosophy department, Glasgow, UK

**Keywords:** Transhumanism, Radical enhancement, Moral status, Identity, Agency, Authenticity

## Abstract

Transhumanism aims to bring about radical human enhancement. In ‘Truly Human Enhancement’ Agar (2014) provides a strong argument against producing radically enhancing effects in agents. This leaves the transhumanist in a quandary—how to achieve radical enhancement whilst avoiding the problem of radically enhancing effects? This paper aims to show that transhumanism can overcome the worries of radically enhancing effects by instead pursuing radical human enhancement via incremental moderate human enhancements (Weak Transhumanism). In this sense, weak transhumanism is much like traditional transhumanism in its aims, but starkly different in its execution. This version of transhumanism is weaker given the limitations brought about by having to avoid radically enhancing effects. I consider numerous objections to weak transhumanism and conclude that the account survives each one. This paper’s proposal of ‘weak transhumanism’ has the upshot of providing a way out of the ‘problem of radically enhancing effects’ for the transhumanist, but this comes at a cost—the restrictive process involved in applying multiple moderate enhancements in order to achieve radical enhancement will most likely be dissatisfying for the transhumanist, however, it is, I contend, the best option available.

## Introduction

The human enhancement debate is for some, centred around the two polarized positions of strong bioconservatism and transhumanism. The former opposes any form of human enhancement, whereas the latter advocates for all possible human enhancements.^1^ However, many philosophers engaged in the continuing debate hold a more nuanced view in favour of some enhancements while rejecting the transhumanist *carte blanche* approach. Some examples of these biomoderate views include A. Buchanan’s (2011) *anti-anti-enhancement* position, I. Persson and J. Savulescu’s (2008) call for research into moral enhancement while rejecting cognitive enhancements and, most recently, N. Agar’s (2014) *truly human enhancement*, where he advocates for some moderate enhancement while rejecting all radical enhancement [8–10]. Agar defines moderate enhancement as improvements in significant attributes and abilities that fall within or just beyond what is currently possible for human beings, and radical enhancements as improvements in significant attributes and abilities that fall far beyond what is currently possible for human beings [10, p. 2].

Agar’s anti-radical enhancement position is bolstered by the many sub-arguments he provides. The first section of this paper will provide a brief overview of these arguments and their conclusions. What follows will be the contention that Agar’s account fails to show why radical enhancement should be avoided; his arguments only present good reason for preventing *radically enhancing effects*.^2^ The aim of this paper is to provide transhumanists with a route to radical enhancement that circumvents the problem of radically enhancing effects. To do this, I present the position of ‘weak transhumanism’ or ‘non-radical radical enhancement,’ which takes Agar’s endorsement of moderate enhancement as a starting point and suggests that this process could well be repeated, involving the addition of moderate enhancements on top of moderate enhancements that would eventually lead to a radical enhancement, from the perspective of the unenhanced. I refer to this version of transhumanism as ‘weak transhumanism’ on the basis that I take it to be a form of transhumanism in its aims but not in its execution.^3^ I grant that many transhumanists will not find weak transhumanism appealing, but I argue it provides the best option if radical enhancement is to be achieved. Following an overview of Agar’s anti-radical enhancement view, along with introducing my own weak transhumanist account, the rest of this paper will consider various objections to the non-radical radical enhancement argument and conclude that my view survives these objections.

## Agar’s anti-radical enhancement position

Agar provides numerous arguments for why pursuing radical enhancement would be ‘rationally imprudent’ for human beings [10]. He claims radical enhancement would result in a transformative change to one’s evaluative framework that could threaten one’s identity. One’s post-enhanced self would no longer relate to one’s past unenhanced experiences and achievements, finding them to be boring and irrelevant.^4^ This fracturing of one’s identity into pre and post enhanced states will effectively kill off one’s past selves.^5^ One’s post-enhanced concerns will be aimed towards the future rather than the mundane and unrelatable past. The memories of one’s past unenhanced life will be more forgettable given their newly perceived insignificance. It would be rationally imprudent to pursue radical enhancement for this very reason.

Further to this point of disconnection between those radically enhanced and those who remain unaltered is the concern that radical enhancement may result in a relative loss of moral status for human beings. ‘Post-persons’^6^ could potentially be justified in elevating their moral status above that of ‘mere-persons,’ given their newfound capacities, resulting in morally acceptable treatment of mere persons that would currently be considered repugnant. Radically enhanced post-persons might be morally justified to treat mere persons like sentient non-humans are currently being treated (medical testing, rescue priority following disasters, etc.) Of course, even with a superior moral status, it does not entail that post-persons will treat mere persons in this manner,^7^ but Agar makes an inductive argument — based on how current humans treat sentient non-humans — that this could well be the case [10]. With this risk in mind, one again sees why pursuing radical enhancement should be considered imprudent and irrational.

Finally, Agar presents a worry that radical enhancement could result in a loss of meaning and enjoyment for mere persons [10]. Radically enhanced scientists will idealize to a more accurate degree than unenhanced scientists. Although progressing humanity closer to the scientific truths of the universe, enhanced scientific idealizations could prove to be beyond the comprehension of unenhanced scientists, who, while recognising the scientific authority of radically enhanced science, will lack any understanding about its conclusions. This lack of understanding would be deeply dissatisfying for unenhanced scientists. Unenhanced scientists would have little grasp of the answers to questions about humanity’s place in the universe and how humanity came to be, and this would impede on unenhanced scientific enquiry. This loss of meaning for the unenhanced will be accompanied by a diminution in engagement and enjoyment as human feats turn into superhuman feats [10]. Agar contends that persons value those feats that fall within current human limits because they can better veridically engage with them, and that those imagined feats of the superhuman fall outside of what they hold valuable, due to their inability to veridically engage with them. Of course, radically enhanced scientists, athletes and spectators may habituate new value(s) from their newly enhanced perspectives, but Agar argues that one should assess whether to pursue radical enhancement from one’s current evaluative framework, not a hypothetical post-person one [10].

Agar’s anti-radical enhancement view should be of serious concern for any transhumanist. However, it is his own endorsement of moderate enhancement that provides a way out for the transhumanist, albeit in a rather prolonged manner and attached to some disappointing concessions.

## Weak transhumanism

Agar’s arguments against radical enhancement are compelling. However, each of his arguments supposes that any would-be radical enhancement is applied to an entirely unenhanced adult population.^8^ With this view, it is obvious that one would be hurtling into the unknown along with the potential losses of identity, value(s), meaning, and moral status among human beings. However, he is not against *all* enhancements and instead advances an argument in favour of moderate enhancement, where the losses would be avoidable given the modest improvement being made on one’s capacities.

Imagine a hypothetical future society of moderately enhanced individuals who have managed to preserve their values, identity, and relative moral status. Once these various moderate enhancements have been in place for some time and the human range of abilities and attributes has been pushed upwards, these new limits will become the new norm. Such a society would be well within its right to moderately enhance itself further again. They could do so with Agar’s concerns in mind. This practice could continue again and again, generation after generation until eventually there would be a moderate enhancement applied that would be considered a radical enhancement from the viewpoint of the unenhanced. In the rest of this paper, I will make the argument that due to this drawn-out process, there seems much less risk of the heavy losses that might otherwise occur should this technical radical enhancement have been utilised by an entirely unenhanced population. Indeed, Agar’s concerns about radical enhancement are only applicable when done *radically.* The steppingstone approach of applying multiple moderate enhancements—over an extended period — to reach what would be a radical enhancement, is a *non-radical* path to radical enhancement. Weak transhumanism, as I am calling it, may not satisfy most transhumanists as they are unlikely to benefit from any radical enhancement given the lengthy route to get there. There would also be restrictions as to when and how many of the enhancements can be provided to an agent; and there is no guarantee that radical enhancement will even be achieved, but it is a way to overcome Agar’s well-voiced objections.

The remainder of this paper will address various objections against the weak transhumanism position being proposed that might be raised by Agar, other biomoderates, and/or the bioconservative. The conclusion reached will be that weak transhumanism survives these objections and provides a more reasonable way to aim for radical enhancement, one that does better than the current ‘no holds barred’ strategy.

## The worry of inauthenticity and diminished agency

A first objection from the bioconservative might raise concerns about the moderately enhancing effects that agents will experience. Although the step-by-step approach to radical enhancement removes the concern about absolute identity splits (that is the obliteration of one’s pre-enhanced self, making way for the formation of one’s post-enhanced self), there remains a worry that some moderate enhancements may still threaten one’s identity in a less absolute fashion by making users feel inauthentic and diminishing their agency [21–23]. This would not create a new species’ psychology – that of a radically enhanced post-human’s - but would alter individual human beings’ original personality suitably enough to appear ‘out of character.’ As a real-world example, recent research into the use of DBS (Deep Brain Stimulation) to treat neurological/psychological disorders ranging from Parkinson’s disease to Obsessive compulsive disorder has shown that some recipients, although benefiting from symptom alleviation, acquire unwanted side-effects of ‘new’ dispositions which can cause them to feel unlike themselves and question what is in fact at the root of their newly acquired trait(s): “Is it them or their device?” [24–26]. This disconcerting feeling can be further bolstered by similar observations from friends and family [27]. This bioconservative rebuttal to the proposal of layered moderate enhancement follows in a similar vein to Agar’s original critique, but note that the above argument likely applies to Agar’s proposal too. An adequate response to the above objection will require one to point out a potential problem with the interpretation of the empirical data while utilising a relational conception of agency. This will be closely linked to the idea of ‘set, setting and matrix’ introduced by researchers of psychedelic experiences [28, 29, 33].

However, there is a stark difference in intent for those undergoing DBS for severe and hindering neurological disorders versus those wishing to neurologically enhance themselves - therapeutics are different than enhancements. Those with Parkinson’s disease who undergo DBS do so with the single goal of alleviating the physical and psychological symptoms of the illness. Felt personality changes were not part of the expected consequences of the treatment. However, any future transhumanist candidate for neurological enhancement would be pursuing such a procedure entirely because of the cognitive and psychological changes it brings, and so is entering into the process better prepared. The importance of the contextual elements of ‘set and setting’ introduced by Leary et al. (1963) to the successful experiences and outcomes of those undergoing psychedelic experiences can be usefully applied here [28]. Focusing first on *set*, which consists of “personality, preparation, expectation, and intention of the person having the experience” [29, p. 1], one can see that the ‘sets’ of those undertaking DBS as therapeutic treatment versus those who might wish to neurologically enhance themselves are vastly different; the latter group are much better positioned to accept and embrace the apparent personality changes that some experience following the procedure. This point does not necessarily undermine the phenomenological experiences of those who have undergone DBS treatment, but it highlights, when interpreting results, the importance of taking into consideration the differing objectives of those applying new technologies to treat disease versus of those those trying to enhance.

Now, this is not to say that feelings of inauthenticity or cases of diminished agency will be avoided. This is yet unknown. But perhaps when changes to one’s baseline capacities and characteristics occur, one can create smoother transitions by understanding agency as relational — both socially and non-socially. The worry that certain enhancement technologies may unduly influence people, without their knowledge, is as relevant for other sources of influence: social media, friends and family, nudge marketing, etc. My agency is made up of these numerous influences [30]. If one accepts that human agency relies or is dependent on others (human and non-human), one can dispose of the worry that certain enhancements might influence users’ actions. Alternatively, the focus should be on how users wish to be influenced, whether the device(s) can grant these wishes, and if the user can oppose those influences [27].

Suggested methods to improve this relationship between users and their enhancements include cultivating ‘agency-competencies’ such as self-reflection, agency practice in new situations, and utilising feedback from friends and family [27]. Or, as T. Brown (2020) refers to these “three skills…: *introspective vigilance, improvisation and relational resourcefulness*” [31, p. 148]. Relational resourcefulness can be extended to include not just those who might observe and comment on negative changes in behaviour, but also experts who can inform users of expected or possible changes. In fact, part of the recommended changes made by Schüpbach et al. (2006) — in the treatment of patients receiving DBS — was to ensure that proper pre-operative and post-operative psychological support was in place for patients to better understand and come to terms with the possible psychological side-effects [32]. Recalling the concept of *setting *— “the physical, social and cultural environment in which the (psychedelic) experience takes place” [29, p. 1] — to show the various aspects of one’s environment that can influence the outcome of a psychedelic experience, Eisner (1997) furthers this concept introducing a third contextual element: that of matrix [33].

Matrix is an extension of the original *setting*, reaching beyond the period of psychedelic experience to include both the pre- and post-session environments of an agent, such as one’s family and living situation. Matrix refers to those aspects of one’s environment that can unduly influence and, in turn, affect one’s agency following a psychedelic experience. Psychedelic research has well-established the importance that the contextual features of set, setting, and matrix have on both the immediate and continued outcome of psychedelic experience [28, 29, 33, 34]. Perhaps adopting a similar conception for other moderately mind-altering technologies/pharmaceuticals would better prepare agents for the psychological and cognitive changes that moderate enhancement aims to bring about.

When considering the above reply to the objection posed by the bioconservative, one can fully appreciate the concerns raised while still providing a framework to navigate through the murky waters of human enhancement technology. For those wishing to be enhanced, ensuring (a) an appropriate *set* is in place prior to the uptake of any enhancement (to which actively seeking cognitive and/or psychological changes would be a contributing factor) and (b) that any changes be limited to a moderate form of enhancement for any one agent given the proposed incremental implementation, then radical changes to an agent’s baseline character are, if the enhancement works as intended, unlikely to occur. In addition, adopting an understanding of agency as being relational in nature, connecting this to an agent’s setting and matrix, and applying the aforementioned skills of ‘introspection vigilance’ and ‘improvisation and relational resourcefulness,’ will better position enhanced agents to avoid feelings of inauthenticity and ensure agency remains intact, if not improved. Note this still leaves open the possibility that feelings of inauthenticity and a diminishment of agency will remain, regardless of efforts to curb such effects. The argument put forward is theoretical and will require supporting empirical data to a degree that is currently unavailable. This will leave the transhumanist on tenterhooks in the interim, but, if the theoretical argument is accurate, it is a way out of the problem as put by the bioconservative.

## The risk of radically enhancing effects (1)

An opponent to the weak transhumanist view has a second objection available — even if a non-radical radical enhancement program was initially effective, there would come a point in time where a new radical enhancement, consisting of the preceding compounded moderate enhancements plus one more, would be applied to a new generation of unenhanced humans and this would virtually act as a radical enhancement, and so Agar’s objections would remain a thorn in the side of the transhumanist project. Overcoming this second objection will require, much to the dismay of the transhumanist, adding three important caveats to the non-radical radical enhancement argument:

**Caveat 1.** For any novel moderate enhancement(s) given to an agent, the resulting enhancing effects should be moderate when compared to the agent’s baseline.

**Caveat 2.** Baseline measurements should be taken from agents at 2 + years.

**Caveat 3.** To avoid a radical enhancing effect from an agent’s baseline, some moderate enhancements must be applied prenatally and/or in an agent’s formative years.

This set of criteria allows for continued moderate enhancement to the point of radical enhancement but avoids the problem of any one agent experiencing a radically enhancing effect.^9^ Allowing multiple moderate enhancements to be applied prenatally and in infancy protects against the potential losses of value(s) and identity that might otherwise occur from doing the same to an unenhanced adult population. Justification for caveat 2 goes as follows: at such an early stage of life only a very basic identity has been formed - a discovery of a subjective self [35–37] - and there is an intuitive incapability to value human feats and scientific endeavour, at least to the degree that Agar argues humans do. The concern that memories from this time would be lost following significant moderate to radical enhancement seems a non-issue when considering the phenomena of childhood amnesia, whereby people, on average, have no memories of their life prior to their 3rd birthday [38, 39]. The memories one holds of one’s self over time plays an important role in one’s sense of personal identity [40], but one’s total forgetting of one’s early childhood, coupled with a rudimentary version of personal identity in those same years overcomes the objections against radical enhancing effects raised by Agar since his worries about a radical enhancement being administered to an unenhanced population presupposes an adult population. In such a case, personal identity is already formed and values instilled. The transformative effects of radically enhancing this mature group put both personal identity and its constituent deeply held value(s) at risk. This is clearly not the case when applying a would-be radical enhancement prenatally and/or during an agents’ infancy. This further restriction to how and when one radically enhances oneself will no doubt come as a blow to the transhumanist. However, it provides a careful pathway through the obstacles laid down by Agar.

Agar might respond to the above reply by accepting that unproblematic radical enhancement could occur for some future generation, but this would leave a mixed society of those radically enhanced and those not, which could result in a relative loss in moral status for those not radically enhanced. As Agar highlights, there exist 3 observable moral statuses:

*Moral status category 1:* The zero moral status possessed by rocks

*Moral status category 2:* The moral status possessed by sentient nonpersons such as sheep and toads.

*Moral status category 3:* The moral status possessed by persons [10, p. 180].

From this, an inductive argument can be made that there could exist a moral status category 4 that would include post-persons. This would leave the unenhanced at risk of a relative loss in moral status and at the receiving end of morally acceptable poorer treatment [42–44].^10^ T. Douglas (2013) offers an interesting hypothetical case of mere persons being used in medical experiments, without consent, in order to aid post-persons [44, p. 474]. Furthermore, both Douglas (2013) and J. McMahan (2009) suggest that moral status could be grounded in persons cognitive and psychological capacity [44, 45]. If such a society did materialise, those with radically improved capacities and attributes may be justified in elevating their moral status above that of mere persons much to the detriment of mere persons. This is a fair point, but one should remember that the non-radical radical enhancement journey consists of many separate moderate enhancements applied over time.^11^ So, a radical enhancement for one particular generation is only radical from the perspective of the unenhanced. For those present at the time, it would be merely another moderate enhancement. The mixed society would consist of those with multiple moderate enhancements and then those with an additional moderate enhancement — this addition resulting in a radical enhancement from the point of view of the unenhanced only.

## The unenhanced and the radically enhanced: worries of a mixed society

The response provided above to the worries of a mixed society raises another potential problem for the non-radical radical enhancement position — what if there are groups who reject all enhancements, much like the Amish community’s general shunning of modern technology? [64]. A similar reaction to enhancement technology would surely leave abstainers worse off in the ways Agar has outlined — their ability to engage in scientific endeavours, their enjoyment of human feats, and their current top tier moral status would all be under threat. If such a group continued to eschew future enhancements while the rest of humanity steadily and repeatedly pushed the limits of (post-) human abilities and attributes upwards, then Agar’s worry of a mixed society of radically enhanced and unenhanced individuals would materialise. The point of the non-radical radical enhancement plan was precisely to avoid such an occurrence and so, if this is the case, then Agar might have weak transhumanism cornered, it now seemingly having returned defensively to square one.

One way to sidestep this issue would be to just bite the bullet. Of course, changes to the fabric of society and make-up of humanity will be divisive. This is true even for Agar’s modest enhancement account. There will always be those resisting the changes that drive humanity forwards, whether it be the luddites of the industrial revolution or the more recent anti-vaccine movement. Although concerns should be aired and the debate had, it would be rather unreasonable to demand that every individual on earth should be convinced of technological proposals prior to their implementation. There was never a global democratic vote in favour of the development and implementation of the internet or mobile telephones or in fact for almost all technological developments, but they are generally accepted by everyone. Furthermore, the weak transhumanist account being proposed would, unlike almost all previous technological developments, call for active democratic participation at each incremental stage.^12^ The benefit of moving forwards with enhancement technologies in a gradual manner is that it allows concerns to be readdressed and the debate to be rehashed prior to each occasion of ‘levelling humanity up.’ This will not be good news for the transhumanist as it leaves open the option for ending the radical enhancement pursuit prior to achieving it. But it allows for a cautiously optimistic approach to radical enhancement, one which grants each new generation a say on humanity’s trajectory.

Opponents to weak transhumanism might question how to proceed if a point is reached where an enhancement with radically enhancing effects is the only available option. The non-radical radical enhancement view entails that all would-be radical enhancements can be reached via incrementally compounding moderate enhancements, but if there is a case where this is not feasible, what decision should be reached about this next possible enhancement? My proposed ‘weak transhumanism’ position forces an unequivocal response: in such a case, *one should not proceed.* As previously acknowledged, Agar provided good reasons against seeking radical enhancement—at least against producing radically enhancing effects in an adult population. To make an exception for radical enhancements where moderate enhancements are unavailable would require one to present a view in favour of ‘strong transhumanism’ which this paper, much like Agar’s, does not endorse. This final restriction on the weak transhumanist venture completes the set of concessions which are required to overcome Agar’s objections and provide an optimistically tentative path towards, eventual, radical enhancement.

It could be argued that given the admission that weak transhumanism could still produce a mixed society of radically enhanced and unenhanced individuals (see the above mentioned Amish case), it would seem markedly unfair to deny those left behind — who then wished to elevate their attributes and capacities to levels within their relative society’s normal range — the option to engage in radical enhancement. I think an exception could be made in those specific cases. Doing so may seem like a contradiction to what I have argued above. However, the argument put forward was against pursuing radical enhancement where no moderate enhancement is available. This option would occur in cases where society was ready to ‘level up’ once more only to find they were unable to produce a next moderate enhancement, instead having only a radical enhancement at their disposal (*radical enhancement as next enhancement*).^13^

The case of an agent undergoing radical enhancement in order to function within that current populations’ normal range is a case where moderate enhancements are available but applying these would leave an agent falling short of the current societal norm (*radical enhancement as catching up*). To endorse cases of ‘radical enhancement as catching up’ intuitively seems fairer for those who fall behind in the enhancement project. But surely Agar’s concerns about radically enhancing unenhanced agents once again apply. At least the risk to mere persons’ identity does. I think one can accept that some risks of radically enhancing unenhanced agents does indeed remain, but these - along with the benefits radical enhancement would bring - can be evaluated on a case-by-case basis. An analogy can be drawn with how certain countries and states conduct legal voluntary euthanasia. For any would-be potential candidate of radical enhancement, a complete assessment of the agent’s physical and psychological health along with a full and proper consent process [47] - where she is made aware of the risks involved - would take place prior to commencing radical enhancement. Weak transhumanism can endorse the case of ‘radical enhancement as catching up’ by showing that in such instances *only* the risk of identity-death remains; the risks of relative loss to moral status and diminishment in engagement in feats and scientific endeavour do not apply in cases where someone is seeking out radical enhancement to meet the new norm of a radically enhanced society. In such cases, the radically enhancing effects produce a gain-in — moral status, capacities for enjoying post-person feats, and engagement in post-person science. Ensuring that certain procedures are followed, the agent in question can rightly choose to ‘psychologically euthanise’ themselves. It is worthwhile to remember that this is purely hypothetical. If such cases were to occur, the decision as to how to proceed would sit with the radically enhanced! In this sense, weak transhumanism would have already achieved its aims. My account acts as a guide towards radical enhancement, but once there, the outcomes become much harder to predict. What I am suggesting may seem rather unsophisticated or completely mistaken to a radically enhanced audience. However, as humanity is not yet radically enhanced, this response seems reasonable and allows for fair instances of radically enhancing unenhanced agents, specific conditions pertaining.

## The risk of radically enhancing effects (2)—tipping points

Concerns might be raised that the repeated application of moderate enhancements to human beings may lead to a ‘tipping point’ of the sort that could lead to a runaway intelligence explosion [48, 49] or some other unexpected radically enhancing effects. For the latter worry, I take the example from climate science and the effects of increasing GHG (greenhouse gases) in the earth’s atmosphere, leading to rising global temperatures which in turn are predicted to lead to the passing of critical climate tipping points, causing radical and irreversible effects on the environment [50].^14^ Regarding the first worry, that of moderate enhancements leading to an intelligence singularity, I think this concern is, for the most part, better aimed at AI technologies, external to the human. Where there exists the possibility of an overlap, I support M. Hutter’s (2016) line of reasoning that “physical and biological limitations likely do not allow singularities,” unless some sophisticated form of mind uploading has occurred [51, p. 275]. But mind uploading would equate to a radical enhancement, and so, since a runaway intelligence explosion would require an initial radical enhancement, the apprehension that moderate enhancements could accidentally cause a radical explosion in intelligence can be put to rest.

What of the second worry — that repeated moderate enhancements might lead to the passing of some currently unknown anthropological tipping point resulting in radical effects?^15^ This could well be the case, but unlike human-caused climate change, weak transhumanism actively seeks to include lengthy interludes between each set of moderate enhancement. The main problem with global warming is that its causes—consumerism, wastefulness, environmental degradation, pollution — are all necessary components of our current social, political, and economic frameworks. Humankind collectively marches towards climate tipping points on a continued hourly basis. This is precisely what makes making the necessary changes to avoid catastrophic climate change so difficult. However, the non-radical radical enhancement project has built-in extended breathing spaces between moderate enhancement stages. If there exists a tipping point(s) that repeated moderate enhancements could lead humanity closer to, the slow and steady approach being advocated for here allows for careful scientific supervision along the way. Along with this expert handholding, weak transhumanism calls for the option to withdraw from the radical enhancement pursuit to be always available. The once in a generation staging of each set of moderate enhancements prevents ‘human enhancement’ from becoming a reliant aspect to the functioning of society at large. This highlighting of the differences between weak transhumanism and anthropological climate change does not eliminate the risk of such a tipping point either existing or, if existing, being passed. However, it shows that the risk is low and can be much better managed than the current problems of human-caused climate change and climate tipping points.

## Humanity’s loss

There is a fifth possible objection that the bioconservative and/or Agar and his supporters might put forward. Suppose that the weak transhumanist idea ends up being a resounding success. The long-drawn-out process of adding multiple moderate enhancements over time ensures that no individual agent experiences radically enhancing effects with the subsequent loss to their baseline set of values, identity, meaning and relative moral status. Furthermore, the achievement is such that almost all individuals partake in the process avoiding the messy consequences of a mix-society of radically enhanced individuals and non-radically enhanced individuals, from the perspective of those alive at that future time. Despite no individual agent experiencing the losses of their values, identity and relative moral status, there will have been a general and historic loss to humanity. The ability to relate to one’s unenhanced ancestors, to appreciate their feats and discoveries and connect to and learn from their experiences will be seriously threatened.

An initial response might be to make the claim that it is not entirely clear why future radically enhanced generations would be incapable of relating to their unenhanced ancestors. They would surely be both capable and interested in their historical journey from mere unenhanced persons to radically enhanced persons. Their appreciation for their unenhanced forebears’ achievements, discoveries and experiences would remain but would merely be qualified — i.e., ‘impressive for an unenhanced person.’ But perhaps this is a poor prediction. Afterall, humanity tends to care more about *Homo sapiens’* history than they do *Homo erectus.’* If radical enhancement resulted in a speciation event, then newly evolved post-persons will have little consideration for mere persons. A second effort at responding to the above objection might want to point to the differences in timescales involved.

Modern humans’ ancestor *Homo erectus’* last appearance on Earth is thought to be, as a latest estimate, 108,000 years ago [52]. Now, if humanity’s evolution from *Homo erectus* to *Homo sapiens* had occurred at both a more rapid rate and more recently, like that of mere persons enhancing themselves to become post-persons, would humanity still be as indifferent to members of that species and their history? Suppose *Homo sapiens* evolved from *Homo erectus* not 500,000–300,000 years ago but just 500–300 years ago. Intuitively, I think one would have, in general, more interest than one currently does for one’s less evolved predecessors. However, more interest does not equate to viewing a 500-year-old member of *Homo erectus* with equal value and consideration as a similar aged *Homo sapiens* person. Any extra consideration one might now give would be purely anthropological/biological in nature. One’s interest in one’s cultural, scientific, and philosophical history would remain squarely with *Homo sapiens*.

These two attempts at overcoming the objection have failed due to the misfiring of the counter response — both aimed to deny the claim that radical enhancement would cause a loss to humanity. A better reply would be to concede that radical enhancement could lead to the creation of post-persons, and that this accelerated evolution of *Homo sapiens* would effectively alienate past unenhanced mere persons from post-persons. However, this loss is not quite the devasting demise of humanity as Agar and others would have one think. Remembering that the whole intention behind the enhancement project is to *improve* human beings. The gradual approach proposed allows for repeated and revisited considerations on the effects that each set of moderate enhancements is having on humanity. Humanity will have to decide repeatedly whether a better existence sits beyond the horizon. The ensuing loss that would occur, if moderate enhancements lead to radical enhancement, would not be caused by the work of some evil misanthrope but rather by the ambitious collective efforts of a humankind pursuing the betterment of its very nature.^16^ The loss in this sense can be framed positively rather than negatively, where the loss is the antecedent to progress and growth. The worry that one would be purposely venturing off into the unknown, with no idea of the consequences, seems less of a concern given the incremental increases being made. There would be a decent idea of what lies ahead on each occasion of moderate enhancement; a slight increase to our baseline attributes and abilities would allow one to veridically engage in those proposed changes. Reframing the loss in this way allows one to accept that ultimate radical enhancement will profoundly alter humanity, but that this altering would be for the bettering of humanity. Furthermore, if this turns out not to be the case — that is, there is evidence the altering leaves humanity worse off — the gradual approach to enhancement being proposed allows for the option to call time on the radical enhancement project.

## What is radical enhancement?

A final objection that could be raised against weak transhumanism points to what might seem like a more fundamental problem. How does one identify a radical enhancement? As more and more moderate enhancements are applied and an eventual radical enhancement manifests, what precisely will have changed to/for the human that allows us to label this totality of enhancements as ‘radical’? Indeed, one might say this is just a new version of the Sorites paradox.^17^ Much like philosophers have asked how many grains of sand it takes to make a heap, so it follows that they might ask how many moderate enhancements it takes to make a radical enhancement. With this in mind, it could be argued all this talk of radical enhancement lacks a conceptual grounding and as such any argument in favour of it ought to be quickly dismissed. If this is correct, this is bad news for weak transhumanism (along with other transhumanist positions) as it fails to overcome this fundamental problem.

A first response can, like Agar, appeal to the intuition one has that there are paradigmatic cases of radical enhancement (e.g., mind uploading, the ability to run at 100mph, telepathy, the capacity to live to be 1000 years-old, etc.). Much like one’s ability to identify heaps without knowing when a heap becomes a heap, one has an intuitive grip on what makes an enhancement radical even if one cannot determine the precise point at which an enhancement becomes radical.

A second response, or rather a fleshing out of the first response, can attempt to introduce certain characteristic features of radical enhancements so as to provide a more concrete idea about how one might go about identifying a radical enhancement in a manner that goes beyond mere intuition. These features should not be thought of as necessary and sufficient conditions, rather they can be used as *indicators* of radical enhancement. When analysing the effects of a particular enhancement, one can utilise these indicators/features as a checklist to assess if the enhancement in question should be considered ‘radical’ or not. An enhancement would not need to qualify for each characteristic feature to be considered radical, rather the meeting of one or more would indicate that the enhancement in question is merely *likely* to be radical. The list below is provided following an in-depth review of the enhancement literature, finding common ground on what characteristic features appear to be in play when discussing radical enhancements. Note, both the transhumanists and bioconservatives directly or indirectly point to these features as being indicative of radical enhancements.

### Feature 1: a new or extended capacity/ability that is an impossibility for any unenhanced human

This feature seems like an obvious indicator of a radical enhancement. Consider the examples mentioned above - mind uploading, the ability to run at 100mph, telepathy, the capacity to live to be 1000 years-old—each of which have been used in the debate as a paradigmatic case of radical enhancement [55, 10, 1, 49, 56]. Each example meets the criteria of feature 1. However, this only *indicates* that the enhancement in question is radical in nature. The reason this feature cannot act as a sufficient condition (it is I think a necessary condition of radical enhancements), is because there are enhancements of the sort that this feature could apply to that intuitively are not radical. For example, consider an enhancement that allows you to vertically jump 10 feet into the air. For reference, the current world record for a vertical jump is 5.4 feet. It seems reasonable to say that such a feat is an impossibility for any unenhanced human, yet nothing about it seems particularly radical.

### Feature 2: a change or high likelihood of change to moral status

As has been previously discussed, radical enhancements are often thought of to be potential moral status changers [42–46]. In most cases the issues raised are about the radically enhanced benefiting from a higher moral status than their unenhanced counterparts. However, it is worthy to note that Buchanan (2009) argues that no such increase in moral status could take place and that J. Gray (2020) argues that radical enhancement could act as a ‘moral status de-enhancer’ [43, 57].^18^

The point here is that any enhancement which leads to a serious debate over the difference in moral status of the enhanced and unenhanced would, regardless of the conclusion reached, likely be a radical enhancement. This feature is neither a necessary nor sufficient condition of radical enhancement. Consider an enhancement which allows one to run 100mph. If this is all that the enhancement can do, there seems to be no grounds for a serious debate around whether the recipient has now had their moral status enhanced, yet it is still intuitively a radical enhancement. A debate may still be had in this example, but it seems entirely baseless to be arguing for any moral status change in virtue of being able to run incredibly fast.

### Feature 3: a significant change in physical and/or cognitive vulnerability

It is widely accepted that one important intention for those pursuing various enhancements of the human being, is to overcome our current vulnerabilities and limitations. Extended healthy lifespans allow one to avoid the ageing process and our death occurring within a 75–85-year range [58]. Mind uploading might accommodate an eternal life of sorts and turn persons into near perfect epistemic agents [49]. Even for the bioconservatives, there is a clear acknowledgement that human enhancements will, or at least aim to, reduce human vulnerability in nearly all its forms: cognitive, physical, existential, and moral [1, 14, 59]. One might think, given the above, it be more fitting that ‘*a significantly reduced vulnerability’* would be the feature that should be used to indicate if a radical enhancement has taken place. However, as M. Coeckelbergh (2013) argues, enhancement technologies and the potential post-human world they bring about, will be laden with many new vulnerabilities [60]. This is not too hard to imagine. Take the development of the modern smartphone as an example — one can now easily ask Google any question one wants to know the answer to, but one can also be hacked and have one’s private information, money, and/or photos stolen by someone on the other side of the world. Both these actions were previously impossible. So, on the one hand, certain limitations have been removed - that is a limit to the sheer amount we can come to know - while on the other, a new vulnerability has been acquired - that one can have personal belongings broken into and their contents stolen by thieves who operate remotely. It is for this reason feature 3 looks for a *significant change* in vulnerability when assessing the results of an enhancement. Where significant change has occurred, this indicates that a radical enhancement is the likely cause.

### Feature 4: an inability for the enhanced and unenhanced to relate to one another

The final feature, which if met would indicate a likely radical enhancement has been utilised, is that it results in a relatability breakdown between the enhanced and unenhanced. This point is nicely captured by Agar (2014) when he argues that radical enhancements would leave the unenhanced unable to ‘veridically engage’ in the superhuman feats of the radically enhanced or allow unenhanced scientists to participate in the research/findings of radically enhanced scientists [10]. In agreement with Agar (2010), B. Davies (2016) highlights a risk to one’s social relationships were some to engage in radical cognitive enhancement [55]. Davies describes the potential tragedy of the enhanced finding their current and past relationships to be ‘meaningless’ and being unable to ‘identify with them at all’ [61, p. 348]. Moreover, R. Sparrow (2013, 2014) and Archer (2016) have questioned if radical moral enhancement might produce parallel societies where the unenhanced are treated as children or worse, like non-human animals [41, 62, 63]. Those in favour of pursuing radical enhancements also capture this inability to relate in their assessment of a future world of enhanced and unenhanced. Bostrom (2008) gives an example of the enhanced developing their own language that allows for more sophisticated communication that ‘unaugmented humans could not even think or experience’ [5, p. 112]. Again, this feature, if in play, points to the likelihood that the enhancement under review is in fact radical, but it does not guarantee it.

The above four features provide a rough checklist that can be used when assessing whether some enhancement should be considered radical. Although feature 1 is a necessary condition for defining radical enhancements, none of the features, singularly or collectively, provide both the necessary and sufficient conditions for defining radical enhancement. Rather, they merely indicate that the enhancement in question should be considered radical in nature.

## Conclusion

The arguments against radical enhancement put forward by Agar were highly convincing and presented a serious problem for the transhumanist. However, Agar’s presupposition that radical enhancement would always be applied to an unenhanced adult population, along with his endorsement for moderate enhancement provides a way for the transhumanist to overcome his skillfully expressed objections. With this assumption in mind, one can see that Agar’s concerns seemed to be directed at *radically enhancing effects* rather than radical enhancement itself. My proposal of weak transhumanism, involving incrementally applying moderate enhancements to future human beings, avoids this problem of agents experiencing radically enhancing effects (except for cases of ‘radical enhancement as catching up,’ but these cases would entail that radical enhancement had already been achieved by society at large). The ‘weakness’ within this transhumanist view comes in the form of various conditions and restrictions: each generation will have a say on the continuation of the radical enhancement project; many enhancements will have to be applied prenatally or within an agent’s formative years; the feasibility of the project will be informed by the science; and only moderate enhancements can be utilised at any incremental stage. Agar highlighted the dangers of pursuing radical enhancement when done *radically*, my proposed gradual pursuit via multiple moderate enhancements is a *non-radical* path towards radical enhancement.

## Footnotes

1 For examples of strong bioconservatism see [1–3]. For examples of transhumanism see N. Bostrom [4–7].

2 To clarify, the weak transhumanism position being presented, although inspired by Agar’s account, is not intended to act as a response *solely* to Agar, rather it aims to position itself within the whole human enhancement debate as a new and better option for transhumanists.

3 Some may have concerns that the focus on enhancements of the human, rather than the tools humans use, is too narrow to be of worry for transhumanists. However, I take it that ‘enhancing the human directly’ is a core aspect of any transhumanist movement, and as such, is alone worthy of debate.

4 For further discussion on enhancement, authenticity and identity see [1, 11–15].

5 P. Crutchfield (2018) reaches the same conclusion when considering the effects of moral enhancement on personal identity [16].

6 Agar’s definition of post-person equates to radically enhanced persons who are ‘beings with moral status higher than persons’ [10, p. 157]. I take Agar to mean that post-persons would belong to a new, highly evolved species of the genus *Homo*. Despite not explicitly being stated, this is what is heavily implied throughout his book. For the sake of this paper post-person(s) will refer to a new species of human whose existence has been brought about through radical enhancement.

7 An interesting comparison can be drawn from animal rights philosophers’ anti-speciesism argument which contends that our poorer treatment of our non-human counterparts is akin to acts of racism and sexism [17, 18]. This is true despite an acknowledged superiority of humans’ capacities. In this sense, post-persons might conclude that mere persons have an equal moral status to them, much like D. DeGrazia (2007) argues Great Apes do to current humans [19, p. 312]. However, the force of Agar’s argument remains. How post-persons might or might not view mere persons is an unknown and therefore his point that there would be a risk of a relative loss in moral status and potential for subsequent poorer treatment holds.

8 When using the term ‘entirely unenhanced’ I take a forward-looking approach from our current position. That is, I acknowledge that there exist well-established and widely accepted forms of enhancement — education, diet, and caffeine intake as examples [20]. But for the sake of discussion, I assume a return to naught. Since these kinds of traditional enhancements are already ingrained within society, they are an entangled part of our current norm. I take Agar to be concerned with new, non-established enhancement technologies/pharmaceuticals. An entirely unenhanced population would be one which has not yet adopted any of these either current non-established or hypothetical new enhancement technologies/pharmaceuticals.

9 A point can be raised here that enhancements will likely produce differing subjective experiences and so it cannot be ruled out that a moderate enhancement could indeed have a radically enhancing effect on an agent due to the individual sensitivities of that agent. However, although the weak transhumanist proposal presented allows for a range of felt experiences from each new set of moderate enhancements — low to low moderate to moderate to high moderate for example — a radically enhancing effect in too many agents would signify that the enhancement in question is *too close* to a radical enhancement to count as being a moderate enhancement. From this all that needs to be conceded is that there may exist a small number of agents who experience radically enhancing effects from some set of moderate enhancements, but that these cases can be thought of much in the same way as medical idiosyncrasies.

10 A. Archer (2016) raises a worry about a mixed society of unenhanced and morally enhanced individuals where the unenhanced would be unable to meet the new standards of praiseworthiness while still, more often than the enhanced, meeting the new standards for blameworthiness. The effect could lead to poorer treatment of the unenhanced in a one of two ways: They would suddenly find themselves blameworthy at a higher rate to current norms or, if they are exempt from these new moral standards, they may be treated like children [41].

11 J. Fabiano (2021) concurs with this paper’s view that ‘*gradual replacement*’ of a person’s naturally endowed psychological capacities with various enhancements over many generations could be a way of creating beings with a higher moral status than that of mere persons while avoiding the harms of abrupt breaks in psychological continuity [46].

12 A full account of the various stakeholders (government, scientists, the most enhanced, the unenhanced, etc.) and their contributions is beyond the scope of this paper.

13 Note this does not rule out possibilities of a future society having both a moderate and radical enhancement available as a next enhancement. In such cases the moderate enhancement should be chosen as use of the radical enhancement would count as an example of ‘radical enhancement as next enhancement.’

14 For the comparison to radical enhancement, I purposely ignore the fact that, if passed, climate tipping points are always bad for human beings, instead focusing on the fact that passing a tipping point can lead to radical changes.

15 Note such radical effects may or may not be *enhancing* in nature.

16 For other discussions surrounding ideas of intentional or enhanced evolution see Bostrom (2003) and J. Harris (2007) [53, 54].

17 Thanks to an anonymous reviewer for both pointing out this similarity and raising this helpful objection.

18 One might think that if an enhancement of some capacity/capacities did indeed result in a de-enhanced moral status, then the enhancement in question is not an enhancement after all. At least not from a holistic point of view.
